# Strong Extension-Free Proof Systems

**DOI:** 10.1007/s10817-019-09516-0

**Published:** 2019-02-22

**Authors:** Marijn J. H. Heule, Benjamin Kiesl, Armin Biere

**Affiliations:** 1 Department of Computer Science, The University of Texas, Austin, USA; 2 Institute of Logic and Computation, TU Wien, Vienna, Austria; 3 CISPA Helmholtz Center i.G., Saarland Informatics Campus, Saarbrücken, Germany; 4 Institute for Formal Models and Verification, Johannes Kepler University, Linz, Austria

**Keywords:** SAT, Propositional proof systems, Proof complexity, Proof checking, Pigeon hole problem, Extended resolution, Clause redundancy

## Abstract

We introduce proof systems for propositional logic that admit short proofs of hard formulas as well as the succinct expression of most techniques used by modern SAT solvers. Our proof systems allow the derivation of clauses that are not necessarily implied, but which are redundant in the sense that their addition preserves satisfiability. To guarantee that these added clauses are redundant, we consider various efficiently decidable redundancy criteria which we obtain by first characterizing clause redundancy in terms of a semantic implication relationship and then restricting this relationship so that it becomes decidable in polynomial time. As the restricted implication relation is based on unit propagation—a core technique of SAT solvers—it allows efficient proof checking too. The resulting proof systems are surprisingly strong, even without the introduction of new variables—a key feature of short proofs presented in the proof-complexity literature. We demonstrate the strength of our proof systems on the famous pigeon hole formulas by providing short clausal proofs without new variables.

## Introduction

Satisfiability (SAT) solvers are used to determine the correctness of hardware and software systems [4, 16]. It is therefore crucial that these solvers justify their claims by providing proofs that can be independently verified. This holds also for various other applications that use SAT solvers. Just recently, long-standing mathematical problems were solved using SAT, including the Erdős Discrepancy Problem [21], the Pythagorean Triples Problem [13], and the computation of the fifth Schur number [10]. Especially in such cases, proofs are at the center of attention and without them the result of a solver is almost worthless. What the mathematical problems and the industrial applications have in common is that proofs are often of considerable size—about 200 terabytes in the case of the Pythagorean Triples Problem and even two petabytes for the fifth Schur number. As the size of proofs is influenced by the strength of their underlying proof system, the search for shorter proofs goes hand in hand with the search for stronger proof systems.

In this article, we introduce highly expressive clausal proof systems that can capture most of the techniques used by modern SAT solvers. Informally, a clausal proof system allows the addition of redundant clauses to a formula in conjunctive normal form (CNF). Here, a clause is considered *redundant* if its addition preserves satisfiability. If the repeated addition of clauses allows us to eventually add the empty clause—which is, by definition, unsatisfiable—then the unsatisfiability of the original formula has been established.

Since the redundancy of clauses is not efficiently decidable in general, practical proof systems only allow the addition of a clause if it fulfills some efficiently decidable criterion that ensures redundancy. For instance, the popular $\mathsf {DRAT}$ proof system [30], which is the de-facto standard in practical SAT solving, only allows the addition of so-called *resolution asymmetric tautologies* [18]. Given a formula and a clause, it can be decided in polynomial time whether the clause is a resolution asymmetric tautology with respect to the formula and therefore the soundness of $\mathsf {DRAT}$ proofs can be checked efficiently.

We present various new redundancy criteria by introducing a characterization of clause redundancy based on a semantic implication relationship between formulas. By replacing the implication relation in this characterization with restricted notions of implication that are computable in polynomial time, we then obtain powerful redundancy criteria that are still efficiently decidable. These redundancy criteria not only generalize earlier ones such as *resolution asymmetric tautologies* [18] or *set-blocked clauses* [19], but they are also closely related to other concepts from the literature, including *autarkies* [24], *safe assignments* [29], *variable instantiation* [1], and *symmetry breaking* [6].

Proof systems based on our new redundancy criteria turn out to be highly expressive, even without allowing the introduction of new variables. This is in contrast to resolution, which is considered relatively weak as long as the introduction of new variables via definitions—as in the stronger proof system of *extended resolution* [9, 26]—is not allowed. The introduction of new variables, however, has a major drawback—the search space of variables and clauses we could possibly add to a proof is clearly exponential. Finding useful clauses with new variables is therefore hard in practice and resulted only in limited success in the past [2, 23].

We illustrate the strength of our strongest proof system by providing short clausal proofs for the famous pigeon hole formulas without introducing new variables. The size of the proofs is linear in the size of the formulas and the clauses added in the proof contain at most two literals. In these proofs, we add redundant clauses that are similar in nature to symmetry-breaking predicates [6, 7]. To verify the correctness of proofs in our new system, we implemented a proof checker. The checker is built on top of DRAT-trim [30], the checker used to validate the unsatisfiability results of the recent SAT competitions [3]. We compare our proofs with existing proofs of the pigeon hole formulas in other proof systems and show that our new proofs are much smaller and cheaper to validate.

This invited article is an extended version of our CADE’17 best paper [14]. Apart from several small improvements throughout the article, we extended the conference version by adding Sect. 7, which describes further interesting properties of the redundant clauses introduced in this article. We also included a new discussion of open problems in Sect. 9.

## Preliminaries

We consider propositional formulas in *conjunctive normal form* (CNF), which are defined as follows. A *literal* is either a variable *x* (a *positive literal*) or the negation $${\overline{x}}$$ of a variable *x* (a *negative literal*). The *complement*$${\overline{l}}$$ of a literal *l* is defined as $${\overline{l}} = {\overline{x}}$$ if $$l = x$$ and $${\overline{l}} = x$$ if $$l = {\overline{x}}$$. For a literal *l*, we denote the variable of *l* by $$ var (l)$$. A *clause* is a disjunction of literals. If not stated otherwise, we assume that clauses do not contain *complementary literals*, i.e., a literal and its complement. A *formula* is a conjunction of clauses. Clauses can be viewed as sets of literals and formulas as sets of clauses. For a set *L* of literals and a formula *F*, we define $$F_L = \{ C \in F \mid C \cap L \ne \emptyset \}$$. We sometimes write $$F_l$$ to denote $$F_{\{l\}}$$.

An *assignment* is a function from a set of variables to the truth values 1 (*true*) and 0 (*false*). An assignment is *total* with respect to a formula if it assigns a truth value to all variables occurring in the formula, otherwise it is *partial*. We often denote assignments by the sequences of literals they satisfy. For instance, $x{\overline{y}}$ denotes the assignment that makes *x* true and *y* false. We denote the domain of an assignment $\alpha$ by $var (\alpha )$. A literal *l* is *satisfied* by an assignment $\alpha$ if *l* is positive and $\alpha ( var (l)) = 1$ or if it is negative and $\alpha ( var (l)) = 0$. A literal is *falsified* by an assignment if its complement is satisfied by the assignment. A clause is satisfied by an assignment $\alpha$ if it contains a literal that is satisfied by $\alpha$. Finally, a formula is satisfied by an assignment $\alpha$ if all its clauses are satisfied by $\alpha$. A formula is *satisfiable* if there exists an assignment that satisfies it. Two formulas are *logically equivalent* if they are satisfied by the same total assignments; they are *satisfiability equivalent* if they are either both satisfiable or both unsatisfiable.

We denote the empty clause by $\bot$ and the satisfied clause by $\top$. Given an assignment $\alpha$ and a clause *C*, we define $C{\,}|{\,}\alpha = \top$ if $\alpha$ satisfies *C*, otherwise $C{\,}|{\,}\alpha$ denotes the result of removing from *C* all the literals falsified by $\alpha$. Moreover, for a formula *F*, we define $F{\,}|{\,}\alpha = \{C{\,}|{\,}\alpha \mid C \in F~\text {and}~ C{\,}|{\,}\alpha \ne \top \}$. We say that a clause *Cblocks* an assignment $\alpha$ if $C = \{x \mid \alpha (x) = 0\} \cup \{{\overline{x}} \mid \alpha (x) = 1\}$. A *unit clause* is a clause that contains only one literal. The result of applying the *unit-clause rule* to a formula *F* is the formula $F{\,}|{\,}\alpha$ with $\alpha$ being an assignment that satisfies a unit clause in *F*. The iterated application of the unit-clause rule to a formula, until no unit clauses are left, is called *unit propagation*. If unit propagation on a formula *F* yields the empty clause $\bot$, we say that it derived a *conflict* on *F*. For example, unit propagation derives a conflict on $F = ({\overline{x}} \vee y) \wedge ({\overline{y}}) \wedge (x)$ since $F{\,}|{\,}x = (y) \wedge ({\overline{y}})$ and $F{\,}|{\,}xy = \bot$.

By $$F \vDash F'$$, we denote that *F* implies $$F'$$, i.e., every assignment that satisfies *F* and assigns all variables in $$ var (F')$$ also satisfies $$F'$$. Furthermore, by $$F \vdash _{^{_{\!\!\!\raisebox {-5pt}{1}}}}F'$$ we denote that for every clause $$(l_1 \vee \dots \vee l_k) \in F'$$, unit propagation derives a conflict on $$F \wedge ({\overline{l}}_1) \wedge \dots \wedge ({\overline{l}}_k)$$. If $$F \vdash _{^{_{\!\!\!\raisebox {-5pt}{1}}}}F'$$, we say that *Fimplies*$$F'$$*via unit propagation*. As an example, $$(x) \wedge (y) \vdash _{^{_{\!\!\!\raisebox {-5pt}{1}}}}(x \vee z) \wedge (y)$$, since unit propagation derives a conflict on both $$(x) \wedge (y) \wedge ({\overline{x}}) \wedge (z)$$ and $$(x) \wedge (y) \wedge ({\overline{y}})$$. Similarly, $$F \vdash _{^{_{\!\!\!\raisebox {-5pt}{0}}}}F'$$ denotes that every clause in $$F'$$ is subsumed by (i.e., is a superset of) a clause in *F*. Observe that $$F \supseteq F'$$ implies $$F \vdash _{^{_{\!\!\!\raisebox {-5pt}{0}}}}F'$$, $$F \vdash _{^{_{\!\!\!\raisebox {-5pt}{0}}}}F'$$ implies $$F \vdash _{^{_{\!\!\!\raisebox {-5pt}{1}}}}F'$$, and $$F \vdash _{^{_{\!\!\!\raisebox {-5pt}{1}}}}F'$$ implies $$F \vDash F'$$.

## Clause Redundancy and Clausal Proofs

In the following, we introduce a formal notion of clause redundancy and demonstrate how it provides the basis for clausal proof systems. We start by introducing clause redundancy [19]:

### Definition 1

A clause *C* is *redundant* with respect to a formula *F* if *F* and $F \wedge C$ are satisfiability equivalent.

For instance, the clause $C = x \vee y$ is redundant with respect to the formula $F = ({\overline{x}} \vee {\overline{y}})$ since *F* and $F \wedge C$ are satisfiability equivalent (although they are not logically equivalent). This redundancy notion allows us to add redundant clauses to a formula without affecting its satisfiability and so it provides the basis for so-called *clausal proof systems*.

In general, given a formula $F = \{C_1, \dots , C_m\}$, a *clausal derivation* of a clause $C_n$ from *F* is a sequence $(C_{m+1},\omega _{m+1}), \dots , (C_n, \omega _n)$ of pairs where $C_i$ is a clause and $\omega _i$, called the *witness*, is a string (for all $i > m$). Such a sequence gives rise to formulas $F_m, F_{m+1}, \dots , F_n$, where $F_i = \{C_1, \dots , C_i\}$. We call $F_i$ the *accumulated formula* corresponding to the *i*th proof step. A clausal derivation is *correct* if every clause $C_i$ ($i > m$) is redundant with respect to the formula $F_{i-1}$ and if this redundancy can be checked in polynomial time (with respect to the size of the proof) using the witness $\omega _i$. A clausal derivation is a *(refutation) proof* of a formula *F* if it derives the empty clause, i.e., if $C_n = \bot$. Clearly, since every clause-addition step preserves satisfiability, and since the empty clause is unsatisfiable, a refutation proof of *F* certifies the unsatisfiability of *F*.

By specifying in detail what kind of redundant clauses—and corresponding witnesses—can be added to a clausal derivation, we obtain concrete proof systems. This is usually done by defining an efficiently checkable syntactic criterion that guarantees that clauses fulfilling this criterion are redundant. A popular example for a clausal proof system is $\mathsf {DRAT}$ [30], the de-facto standard for unsatisfiability proofs in practical SAT solving. $\mathsf {DRAT}$ allows the addition of a clause if it is a so-called *resolution asymmetric tautology* [18] ($\mathsf {RAT}$, defined in the next section). As it can be efficiently checked whether a clause is a $\mathsf {RAT}$ with respect to a formula, and since $\mathsf {RAT}$s cover many types of redundant clauses, the $\mathsf {DRAT}$ proof system is very powerful.

The strength of a clausal proof system depends on the *generality* of the underlying redundancy criterion. We say that a redundancy criterion ${{\mathcal {R}}}_1$ is *more general* than a redundancy criterion ${{\mathcal {R}}}_2$ if, whenever ${{\mathcal {R}}}_2$ identifies a clause *C* as redundant with respect to a formula *F*, then ${{\mathcal {R}}}_1$ also identifies *C* as redundant with respect to *F*. For instance, whenever a clause is subsumed in some formula, it is a $\mathsf {RAT}$ with respect to that formula. Therefore, the $\mathsf {RAT}$ redundancy criterion is more general than the subsumption criterion. In the next section, we develop redundancy criteria that are even more general than $\mathsf {RAT}$, thus giving rise to proof systems that are stronger than $\mathsf {DRAT}$.

## Clause Redundancy via Implication

In the following, we introduce a characterization of clause redundancy that reduces the question whether a clause is redundant with respect to a certain formula to a simple question of implication. The advantage of this is that we can replace the logical implication relation by polynomially decidable implication relations to derive powerful redundancy criteria that are still efficiently checkable. These redundancy criteria can then be used to obtain highly expressive clausal proof systems.

Our characterization is based on the observation that a clause in a CNF formula can be seen as a constraint that blocks those assignments that falsify the clause. Therefore, a clause can be safely added to a formula if it does not constrain the formula too much. What we mean by this is that after adding the clause, there should still exist other assignments (i.e., assignments not blocked by the clause) under which the formula is at least as satisfiable as under the assignments blocked by the clause. Consider the following example:

### Example 1

Let $F = (x \vee y) \wedge (x \vee z) \wedge ({\overline{x}} \vee y \vee z)$ and consider the (unit) clause $C = x$ which blocks all assignments that falsify *x*. The addition of *C* to *F* does not affect satisfiability: Let $\alpha = {\overline{x}}$ and $\omega = x$. Then, $F{\,}|{\,}\alpha = (y) \wedge (z)$ while $F{\,}|{\,}\omega = (y \vee z)$. Clearly, every satisfying assignment of $F{\,}|{\,}\alpha$ is also a satisfying assignment of $F{\,}|{\,}\omega$, i.e., $F{\,}|{\,}\alpha \vDash F{\,}|{\,}\omega$. Thus, *F* is at least as satisfiable under $\omega$ as it is under $\alpha$. Moreover, $\omega$ satisfies *C*. The addition of *C* does therefore not affect the satisfiability of *F*. $\square$

This motivates our new characterization of clause redundancy presented next. The characterization requires the existence of an assignment that satisfies the clause and so it is only applicable to non-empty clauses. Note that for a given clause *C*, “*the* assignment $\alpha$ blocked by *C*”, as defined above in Sect. 2, is in general a partial assignment and thus *C* actually rules out all assignments that extend $\alpha$:

### Theorem 1

Let *F* be a formula, *C* a non-empty clause, and $\alpha$ the assignment blocked by *C*. Then, *C* is redundant with respect to *F* if and only if there exists an assignment $\omega$ such that $\omega$ satisfies *C* and $F {\,}|{\,}\alpha \vDash F{\,}|{\,}\omega$.

### Proof

For the “only if” direction, assume that *F* and $F \wedge C$ are satisfiability equivalent. If $F{\,}|{\,}\alpha$ is unsatisfiable, then $F{\,}|{\,}\alpha \vDash F{\,}|{\,}\omega$ for every $\omega$, hence the statement trivially holds. Assume now that $F{\,}|{\,}\alpha$ is satisfiable, implying that *F* is satisfiable. Then, since *F* and $F \wedge C$ are satisfiability equivalent, there exists an assignment $\omega$ that satisfies both *F* and *C*. Thus, since $\omega$ satisfies *F*, it holds that $F{\,}|{\,}\omega = \emptyset$ and so $F{\,}|{\,}\alpha \vDash F{\,}|{\,}\omega$.

For the “if” direction, assume that there exists an assignment $\omega$ such that $\omega$ satisfies *C* and $F{\,}|{\,}\alpha \vDash F {\,}|{\,}\omega$. Now, let $\gamma$ be a (total) assignment that satisfies *F* and falsifies *C*. We show how $\gamma$ can be turned into a satisfying assignment $\gamma '$ of $F \wedge C$. As $\gamma$ falsifies *C*, it coincides with $\alpha$ on $var (\alpha )$. Therefore, since $\gamma$ satisfies *F*, it must satisfy $F{\,}|{\,}\alpha$ and since $F{\,}|{\,}\alpha \vDash F{\,}|{\,}\omega$ it must also satisfy $F{\,}|{\,}\omega$. Now, consider the following assignment:

$$\begin{aligned} \gamma '(x) = {\left\{ \begin{array}{ll} \omega (x) &{}\text {if}~{ x \in var (\omega ),}\\ \gamma (x) &{}\text {otherwise.} \end{array}\right. } \end{aligned}$$

Clearly, since $\omega$ satisfies *C*, $\gamma '$ also satisfies *C*. Moreover, as $\gamma$ satisfies $F{\,}|{\,}\omega$ and $var (F{\,}|{\,}\omega ) \subseteq var (\gamma ) \setminus var (\omega )$, $\gamma '$ satisfies *F*. Hence, $\gamma '$ satisfies $F \wedge C$. $\square$

This alternative characterization of redundancy allows us to replace the logical implication relation by restricted implication relations that are polynomially decidable. For instance, we can replace the condition $F{\,}|{\,}\alpha \vDash F {\,}|{\,}\omega$ by the restricted condition $F{\,}|{\,}\alpha \vdash _{^{_{\!\!\!\raisebox {-5pt}{1}}}}F{\,}|{\,}\omega$ (likewise, we could also use relations such as “$\vdash _{^{_{\!\!\!\raisebox {-5pt}{0}}}}$” or “$\supseteq$” instead of “ $\vdash _{^{_{\!\!\!\raisebox {-5pt}{1}}}}$”). Now, if we are given a clause *C*—which implicitly gives us the blocked assignment $\alpha$—and a *witnessing assignment* $\omega$, then we can check in polynomial time whether $F{\,}|{\,}\alpha \vdash _{^{_{\!\!\!\raisebox {-5pt}{1}}}}F{\,}|{\,}\omega$, which is a sufficient condition for the redundancy of *C* with respect to *F*. We can therefore use this implication-based redundancy notion to define proof systems. The witnessing assignments can then be used as witnesses in the proof.

**Fig. 1:**
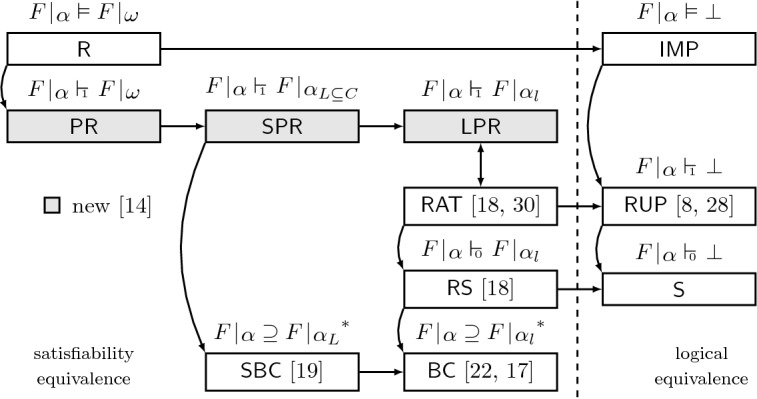
Landscape of redundancy notions of non-empty clauses. $\mathsf {R}$ denotes all redundant clauses and $\mathsf {IMP}$ stands for implied clauses. A path from *X* to *Y* indicates that *X* is more general than *Y*. The asterisk ($^*$) denotes that the exact characterization implies the shown one, e.g., for every set-blocked clause, the property $F{\,}|{\,}\alpha \supseteq F{\,}|{\,}\alpha _L$ holds, but not vice versa

In the following, we use the propagation-implication relation “ $\vdash _{^{_{\!\!\!\raisebox {-5pt}{1}}}}$” to define the redundancy criteria of 
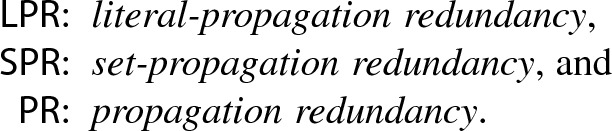
 Basically, the three notions differ in the way we allow the witnessing assignment $\omega$ to differ from the assignment $\alpha$ blocked by a clause. The more freedom we give to $\omega$, the more general the redundancy notion we obtain. We show that $\mathsf {LPR}$ clauses—the least general of the three—coincide with $\mathsf {RAT}$. For the more general $\mathsf {SPR}$ clauses, we show that they generalize set-blocked clauses ($\mathsf {SBC}$) [19], which is not the case for $\mathsf {LPR}$ clauses. Finally, $\mathsf {PR}$ clauses are the most general ones. They give rise to an extremely powerful proof system. The new landscape of redundancy notions we thereby obtain is illustrated in Fig. 1. In the figure, $\mathsf {RUP}$ stands for the redundancy notion based on reverse unit propagation [8, 28], $\mathsf {S}$ stands for subsumed clauses, $\mathsf {RS}$ for clauses with subsumed resolvents [18], and $\mathsf {BC}$ for blocked clauses [17, 22].

As we will see, when defining proof systems based on $\mathsf {LPR}$ (e.g., the $\mathsf {DRAT}$ system) or $\mathsf {SPR}$ clauses, we do not need to explicitly add the redundancy witnesses (i.e., the witnessing assignments $\omega$) to a proof. Thus, $\mathsf {LPR}$ and $\mathsf {SPR}$ proofs can just be seen as a sequence of clauses. In particular, a proof system based on $\mathsf {SPR}$ clauses can have the same syntax as $\mathsf {DRAT}$ proofs, which makes it “downwards compatible”. This is in contrast to proof systems based on $\mathsf {PR}$ clauses, where in general witnessing assignments have to be added to a proof. Otherwise redundancy of a clause can not be checked in polynomial time.

We start by introducing $\mathsf {LPR}$ clauses. In the following, given a (partial) assignment $\alpha$ and a set *L* of literals, we denote by $\alpha _L$ the assignment obtained from $\alpha$ by making all literals in *L* true. If *L* contains only a single literal, we sometimes write $\alpha _l$ to denote $\alpha _{\{l\}}$. In the conference paper [14], we used a slightly different definition, saying that $\alpha _L$ is obtained from $\alpha$ by flipping the truth values of all literals in *L*. Since we only defined $\alpha _L$ for assignments $\alpha$ that falsify all the literals in *L*, nothing changes. We do, however, believe that the new notion is more intuitive.

### Definition 2

Let *F* be a formula, *C* a clause, and $\alpha$ the assignment blocked by *C*. Then, *C* is *literal-propagation redundant* ($\mathsf {LPR}$) with respect to *F* if there exists a literal $l \in C$ such that $F{\,}|{\,}\alpha \vdash _{^{_{\!\!\!\raisebox {-5pt}{1}}}}F{\,}|{\,}\alpha _l$.

### Example 2

Let $F = (x \vee y)\wedge (x \vee {\overline{y}} \vee z) \wedge ({\overline{x}} \vee z)$ and let *C* be the unit clause *x*. Then, $\alpha = {\overline{x}}$ is the assignment blocked by *C*, and $\alpha _x = x$. Now, consider $F{\,}|{\,}\alpha = (y) \wedge ({\overline{y}} \vee z)$ and $F{\,}|{\,}\alpha _x = (z)$. Clearly, $F{\,}|{\,}\alpha \vdash _{^{_{\!\!\!\raisebox {-5pt}{1}}}}F{\,}|{\,}\alpha _x$ and therefore *C* is literal-propagation redundant with respect to *F*. $\square$

The $\mathsf {LPR}$ definition is quite restrictive since it requires the witnessing assignment $\alpha _l$ to disagree with $\alpha$ on exactly one variable. Nevertheless, this already suffices for $\mathsf {LPR}$ clauses to coincide with $\mathsf {RAT}$s [18]:

### Definition 3

Let *F* be a formula and *C* a clause. Then, *C* is a *resolution asymmetric tautology* ($\mathsf {RAT}$) with respect to *F* if there exists a literal $l \in C$ such that, for every clause $D \in F_{{\overline{l}}}$, $F \vdash _{^{_{\!\!\!\raisebox {-5pt}{1}}}}C \cup (D \setminus \{{\overline{l}}\})$.

### Theorem 2

A clause *C* is $\mathsf {LPR}$ with respect to a formula *F* if and only if it is a $\mathsf {RAT}$ with respect to *F*.

### Proof

For the “only if” direction, assume that *C* is $\mathsf {LPR}$ with respect to *F*, i.e., *C* contains a literal *l* such that $F{\,}|{\,}\alpha \vdash _{^{_{\!\!\!\raisebox {-5pt}{1}}}}F{\,}|{\,}\alpha _l$. Now, let $D \in F_{{\overline{l}}}$. We have to show that $F \vdash _{^{_{\!\!\!\raisebox {-5pt}{1}}}}C \cup (D \setminus \{{\overline{l}}\})$. First, note that $F{\,}|{\,}\alpha$ is exactly the result of propagating the negated literals of *C* on *F*, i.e., applying the unit-clause rule with the negated literals of *C* but not performing further propagations. Moreover, since $\alpha _l$ falsifies ${\overline{l}}$, it follows that $D{\,}|{\,}\alpha _l \subseteq (D \setminus \{{\overline{l}}\})$. But then, since $F{\,}|{\,}\alpha \vdash _{^{_{\!\!\!\raisebox {-5pt}{1}}}}D{\,}|{\,}\alpha _l$, it must hold that $F \vdash _{^{_{\!\!\!\raisebox {-5pt}{1}}}}C \cup (D \setminus \{{\overline{l}}\})$, hence *C* is a $\mathsf {RAT}$ with respect to *F*.

For the “if” direction, assume that *C* is a $\mathsf {RAT}$ with respect to *F*, i.e., *C* contains a literal *l* such that, for every clause $D \in F_{{\overline{l}}}$, $F \vdash _{^{_{\!\!\!\raisebox {-5pt}{1}}}}C \cup (D \setminus \{{\overline{l}}\})$. Now, let $D{\,}|{\,}\alpha _l \in F{\,}|{\,}\alpha _l$ for $D \in F$. We have to show that $F{\,}|{\,}\alpha \vdash _{^{_{\!\!\!\raisebox {-5pt}{1}}}}D{\,}|{\,}\alpha _l$. Since $\alpha _l$ satisfies *l* and $\alpha$ falsifies *C*, *D* does neither contain *l* nor any negations of literals in *C* except for possibly ${\overline{l}}$. If *D* does not contain ${\overline{l}}$, then $D{\,}|{\,}\alpha = D{\,}|{\,}\alpha _l$ is contained in $F{\,}|{\,}\alpha$ and hence the claim immediately follows.

Assume therefore that ${\overline{l}} \in D$. As argued for the other direction, propagating the negated literals of *C* (and no other literals) on *F* yields $F{\,}|{\,}\alpha$. Therefore, since $F \vdash _{^{_{\!\!\!\raisebox {-5pt}{1}}}}C \cup (D \setminus \{{\overline{l}}\})$ and $D \setminus \{{\overline{l}}\}$ does not contain any negations of literals in *C* (which could otherwise be the reason for a unit propagation conflict that only happens because of *C* containing a literal whose negation is contained in $D \setminus \{{\overline{l}}\}$), it must be the case that $F{\,}|{\,}\alpha \vdash _{^{_{\!\!\!\raisebox {-5pt}{1}}}}D \setminus \{{\overline{l}}\}$. Now, the only literals of $D \setminus \{{\overline{l}}\}$ that are not contained in $D{\,}|{\,}\alpha _l$ are the ones falsified by $\alpha$, but those are anyhow not contained in $F{\,}|{\,}\alpha$. Hence, $F{\,}|{\,}\alpha \vdash _{^{_{\!\!\!\raisebox {-5pt}{1}}}}D{\,}|{\,}\alpha _l$ and thus *C* is $\mathsf {LPR}$ with respect to *F*. $\square$

By allowing the witnessing assignments to disagree with $\alpha$ on more than only one literal, we obtain the more general notion of set-propagation-redundant clauses, which we introduce next. In the following, for a set *L* of literals, we define ${\bar{L}} = \{{\overline{l}} \mid l \in L\}$.

### Definition 4

Let *F* be a formula, *C* a clause, and $\alpha$ the assignment blocked by *C*. Then, *C* is *set-propagation redundant* ($\mathsf {SPR}$) with respect to *F* if there exists a non-empty set $L \subseteq C$ of literals such that $F{\,}|{\,}\alpha \vdash _{^{_{\!\!\!\raisebox {-5pt}{1}}}}F{\,}|{\,}\alpha _L$.

### Example 3

Let $F = ( x \vee y) \wedge (x \vee {\overline{y}} \vee z) \wedge ({\overline{x}} \vee z) \wedge ({\overline{x}} \vee u) \wedge ({\overline{u}} \vee x)$, $C = x \vee u$, and $L = \{x, u\}$. Then, $\alpha = \overline{x}\overline{u}$ is the assignment blocked by *C*, and $\alpha _L = xu$. Now, consider $F{\,}|{\,}\alpha = (y) \wedge ({\overline{y}} \vee z)$ and $F{\,}|{\,}\alpha _L = (z)$. Clearly, $F{\,}|{\,}\alpha \vdash _{^{_{\!\!\!\raisebox {-5pt}{1}}}}F{\,}|{\,}\alpha _L$ and so *C* is set-propagation redundant with respect to *F*. Note also that *C* is not literal-propagation redundant with respect to *F*.

Since *L* is a subset of *C*, we do not need to add it (or the assignment $\alpha _L$) explicitly to an $\mathsf {SPR}$ proof. By requiring that *L* must consist of the first literals of *C* when adding *C* to a proof (viewing a clause as a sequence of literals), we can ensure that the $\mathsf {SPR}$ property is efficiently decidable. For instance, when a proof contains the clause $l_1 \vee \dots \vee l_k$, we first check whether the $\mathsf {SPR}$ property holds under the assumption that $L = \{l_1\}$. If not, we proceed by assuming that $L = \{l_1, l_2\}$, and so on until $L = \{l_1, \dots , l_k\}$. Thereby, only linearly many candidates for *L* need to be checked. In contrast to $\mathsf {LPR}$ clauses and $\mathsf {RAT}$s, the notion of $\mathsf {SPR}$ clauses generalizes set-blocked clauses [19]:

### Definition 5

A clause *C* is *set-blocked* ($\mathsf {SBC}$) by a non-empty set $L \subseteq C$ in a formula *F* if, for every clause $D \in F_{{{\bar{L}}}}$, the clause $(C \setminus L) \cup {\bar{L}} \cup D$ contains two complementary literals.

To show that set-propagation-redundant clauses generalize set-blocked clauses, we first characterize them as follows:

### Lemma 3

Let *F* be a clause, *C* a formula, $L \subseteq C$ a non-empty set of literals, and $\alpha$ the assignment blocked by *C*. Then, *C* is set-blocked by *L* in *F* if and only if, for every $D \in F$, $D{\,}|{\,}\alpha = \top$ implies $D{\,}|{\,}\alpha _L = \top$.

### Proof

For the “only if” direction, assume that there exists a clause $D \in F$ such that $D{\,}|{\,}\alpha = \top$ but $D{\,}|{\,}\alpha _L \ne \top$. Then, since $\alpha$ and $\alpha _L$ disagree only on literals in *L*, it follows that *D* contains a literal $l \in {{\bar{L}}}$ and thus $D \in F_{{{\bar{L}}}}$. Now, $\alpha _L$ falsifies exactly the literals in $(C \setminus L) \cup {{\bar{L}}}$ and since it does not satisfy any of the literals in *D*, it follows that there exists no literal $l \in D$ such that its complement ${\overline{l}}$ is contained in $(C \setminus L) \cup {{\bar{L}}}$. Therefore, *C* is not $\mathsf {SBC}$ by *L* in *F*.

For the “if” direction, assume that *C* is not $\mathsf {SBC}$ by *L* in *F*, i.e., there exists a clause $D \in F_{{{\bar{L}}}}$ such that $(C \setminus L) \cup {{\bar{L}}} \cup D$ does not contain complementary literals. Now, $D{\,}|{\,}\alpha = \top$ since $\alpha$ falsifies *L* and $D \cap {{\bar{L}}} \ne \emptyset$. Since *D* contains no literal *l* such that ${\overline{l}} \in (C \setminus L) \cup {{\bar{L}}}$ and since $\alpha _L$ falsifies exactly the literals in $(C \setminus L) \cup {{\bar{L}}}$, it follows that $\alpha _L$ does not satisfy *D*, hence $D{\,}|{\,}\alpha _L \ne \top$. $\square$

### Theorem 4

If a clause *C* is set-blocked by a set *L* in a formula *F*, it is set-propagation redundant with respect to *F*.

### Proof

Assume that *C* is set-blocked by *L* in *F*. We show that $F{\,}|{\,}\alpha \supseteq F{\,}|{\,}\alpha _L$, which implies that $F{\,}|{\,}\alpha \vdash _{^{_{\!\!\!\raisebox {-5pt}{1}}}}F{\,}|{\,}\alpha _L$, and therefore that *C* is set-propagation redundant with respect to *F*. Let $D{\,}|{\,}\alpha _L \in F{\,}|{\,}\alpha _L$. First, note that *D* cannot be contained in $F_L$, for otherwise $D{\,}|{\,}\alpha _L = \top$ and thus $D{\,}|{\,}\alpha _L \notin F{\,}|{\,}\alpha _L$. Second, observe that *D* can also not be contained in $F_{{{\bar{L}}}}$, since that would imply that $D{\,}|{\,}\alpha = \top$ and thus, by Lemma 3, $D{\,}|{\,}\alpha _L = \top$. Therefore, $D \notin F_L \cup F_{{{\bar{L}}}}$ and so $D{\,}|{\,}\alpha = D{\,}|{\,}\alpha _L$. But then, $D{\,}|{\,}\alpha _L \in F{\,}|{\,}\alpha$. It follows that $F{\,}|{\,}\alpha \supseteq F{\,}|{\,}\alpha _L$. $\square$

We thus know that set-propagation-redundant clauses generalize both resolution asymmetric tautologies and set-blocked clauses. As there are resolution asymmetric tautologies that are not set-blocked (and vice versa) [19], it follows that set-propagation-redundant clauses are actually a *strict* generalization of these two kinds of clauses.

Note that $F{\,}|{\,}\alpha \vdash _{^{_{\!\!\!\raisebox {-5pt}{1}}}}F{\,}|{\,}\alpha _L$ is equivalent to $F{\,}|{\,}\alpha \vdash _{^{_{\!\!\!\raisebox {-5pt}{1}}}}F_{{{\bar{L}}}}{\,}|{\,}\alpha _L$. To see this, observe that if a clause $D{\,}|{\,}\alpha _L \in F{\,}|{\,}\alpha _L$ contains no literals from ${{\bar{L}}}$, then $\alpha _L$ does not assign any of its literals, in which case $D{\,}|{\,}\alpha _L$ is also contained in $F{\,}|{\,}\alpha$. We therefore do not need to check for every $D{\,}|{\,}\alpha _L \in F{\,}|{\,}\alpha _L$ whether $F{\,}|{\,}\alpha \vdash _{^{_{\!\!\!\raisebox {-5pt}{1}}}}D{\,}|{\,}\alpha$.

By giving practically full freedom to the witnessing assignments, i.e., by only requiring them to satisfy *C*, we finally arrive at propagation-redundant clauses, the most general of the three redundancy notions:

### Definition 6

Let *F* be a formula, *C* a clause, and $\alpha$ the assignment blocked by *C*. Then, *C* is *propagation redundant* ($\mathsf {PR}$) with respect to *F* if there exists an assignment $\omega$ such that $\omega$ satisfies *C* and $F{\,}|{\,}\alpha \vdash _{^{_{\!\!\!\raisebox {-5pt}{1}}}}F{\,}|{\,}\omega$.

### Example 4

Let $F = (x \vee y) \wedge ({\overline{x}} \vee y) \wedge ({\overline{x}} \vee z)$, $C = x$, and let $\omega = xz$ be the witnessing assignment. Then, $\alpha = {\overline{x}}$ is the assignment blocked by *C*. Now, consider $F{\,}|{\,}\alpha = (y)$ and $F{\,}|{\,}\omega = (y)$. Clearly, unit propagation with the negated literal ${\overline{y}}$ of the unit clause $y \in F{\,}|{\,}\omega$ derives a conflict on $F{\,}|{\,}\alpha$. Therefore, $F{\,}|{\,}\alpha \vdash _{^{_{\!\!\!\raisebox {-5pt}{1}}}}F{\,}|{\,}\omega$ and so *C* is $\mathsf {PR}$ with respect to *F*. Note that *C* is not set-propagation redundant because for $L = \{x\}$, we have $\alpha _L =x$ and so $F{\,}|{\,}\alpha _L$ contains the two unit clauses *y* and *z*, but it does not hold that $F{\,}|{\,}\alpha \vdash _{^{_{\!\!\!\raisebox {-5pt}{1}}}}z$. The fact that $\omega$ satisfies *z* is crucial for ensuring propagation redundancy. $\square$

Since the witnessing assignments $\omega$ are allowed to assign variables that are not contained in *C*, we need—at least in general—to add them to a proof to guarantee that redundancy can be efficiently checked.

We can now explicitly define the $\mathsf {PR}$ proof system as an instance of a clausal proof system as defined on page 4:

### Definition 7

Given a formula $F = \{C_1, \dots , C_m\}$, a $\mathsf {PR}$*derivation* of a clause $C_n$ from *F* is a sequence $(C_{m+1},\omega _{m+1}),\dots , (C_n,\omega _n)$ where for every pair $(C_i, \omega _i)$, one of the following holds: (1) $\omega _i$ is an assignment that satisfies $C_i$ and $F_{i-1}{\,}|{\,}\alpha _i \vdash _{^{_{\!\!\!\raisebox {-5pt}{1}}}}F_{i-1}{\,}|{\,}\omega _i$ with $\alpha _i$ being the assignment blocked by $C_i$, or (2) $C_n = \bot$ and $F_{n-1} \vdash _{^{_{\!\!\!\raisebox {-5pt}{1}}}}\bot$. A $\mathsf {PR}$ derivation of $\bot$ from *F* is a $\mathsf {PR}$*proof* of *F*.

The $\mathsf {LPR}$ proof system and the $\mathsf {SPR}$ proof system are defined accordingly. Note that in the definition above we treat the empty clause separately because only non-empty clauses can be propagation redundant. If we allow the mentioned proof systems to delete arbitrary clauses, we obtain the proof systems $\mathsf {D}\mathsf {LPR}$, $\mathsf {D}\mathsf {SPR}$, and $\mathsf {D}\mathsf {PR}$. We will not consider deletion in the rest of the article.

## Short Proofs of the Pigeon Hole Principle

In a landmark article, Haken [9] showed that pigeon hole formulas cannot be refuted by resolution proofs that are of polynomial size with respect to the size of the formulas. In contrast, Cook [5] proved that there are actually polynomial-size refutations of the pigeon hole formulas in the stronger proof system of *extended resolution*. What distinguishes extended resolution from general resolution is that it allows the introduction of new variables via definitions. Cook showed how the introduction of such definitions helps to reduce a pigeon hole formula of size *n* to a pigeon hole formula of size $n-1$ over new variables. The problem with the introduction of new variables, however, is that the search space of possible variables—and therefore clauses—that could be added to a proof is exponential.

In the following, we illustrate how the $$\mathsf {PR}$$ proof system admits short proofs of pigeon hole formulas without the need for introducing new variables. This shows that the $$\mathsf {PR}$$ system is strictly stronger than the resolution calculus, even when we forbid the introduction of new variables. A pigeon hole formula $$ PHP _{n}$$ intuitively encodes that $$n + 1$$ pigeons have to be assigned to *n* holes such that no hole contains more than one pigeon.^1^ In the encoding, a variable $$x_{p,h}$$ intuitively denotes that pigeon *p* is assigned to hole *h*:

$$\begin{aligned} PHP _{n} := \bigwedge _{1 \le p \le n + 1} (x_{p,1} \vee \dots \vee x_{p,n}) \wedge \bigwedge _{1 \le p < q \le n + 1}\bigwedge _{1 \le h \le n} ({\overline{x}}_{p,h} \vee {\overline{x}}_{q,h}) \end{aligned}$$

Clearly, pigeon hole formulas are unsatisfiable. The main idea behind our approach is similar to that of Cook, namely to reduce a pigeon hole formula $$ PHP _{n}$$ to the smaller $$ PHP _{n-1}$$. The difference is that in our case $$ PHP _{n-1}$$ is still defined on the same variables as $$ PHP _{n}$$. Therefore, reducing $$ PHP _{n}$$ to $$ PHP _{n-1}$$ boils down to deriving the clauses $$x_{p,1} \vee \dots \vee x_{p,n-1}$$ for $$1 \le p \le n$$.

Following Haken [9], we use array notation for clauses: Every clause is represented by an array of $$n + 1$$ columns and *n* rows. An array contains a “” (“”) in the *p*th column and *h*th row if and only if the variable $$x_{p,h}$$ occurs positively (negatively, respectively) in the corresponding clause. Representing $$ PHP _{n}$$ in array notation, we have for every clause $$x_{p,1} \vee \dots \vee x_{p,n}$$, an array in which the *p*th column is filled with “”. Moreover, for every clause $${\overline{x}}_{p,h} \vee {\overline{x}}_{q,h}$$, we have an array that contains two “” in row *h*—one in column *p* and the other in column *q*. For instance, $$ PHP _{3}$$ is given in array notation as follows: 
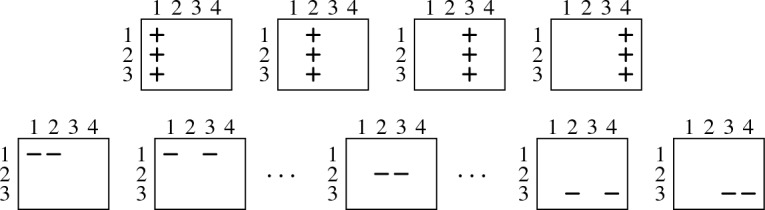
 We illustrate the general idea for reducing a pigeon hole formula $$ PHP _{n}$$ to the smaller $$ PHP _{n-1}$$ on the concrete formula $$ PHP _{3}$$. It should, however, become clear from our explanation that the procedure works for every $$n > 1$$. If we want to reduce $$ PHP _{3}$$ to $$ PHP _{2}$$, we have to derive the following three clauses: 
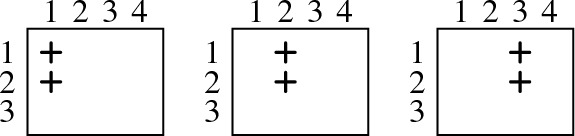
 We can do so by removing the “” from the last row of every column full of “”, except for the last column, which can be ignored as it is not contained in $$ PHP _{2}$$. The key observation is that a “” in the last row of the *p*th column can be removed with the help of so-called “diagonal clauses” of the form $${\overline{x}}_{p,n} \vee {\overline{x}}_{n+1,h}$$ ($$1 \le h \le n-1$$). We are allowed to add these diagonal clauses since they are, as we will show, propagation redundant with respect to $$ PHP _{n}$$. The arrays below represent the diagonal clauses to remove the “” from the last row of the first (left), second (middle), and third column (right): 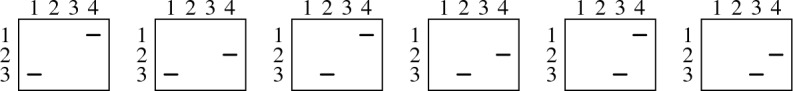 We next show how exactly these diagonal clauses allow us to remove the bottom “” from a column full of “”, or, in other words, how they help us to remove the literal $$x_{p,n}$$ from a clause $$x_{p,1} \vee \dots \vee x_{p,n}$$ ($$1 \le p \le n$$). Consider, for instance, the clause $$x_{2,1} \vee x_{2,2} \vee x_{2,3}$$ in $$ PHP _{3}$$. Our aim is to remove the literal $$x_{2,3}$$ from this clause. Before we explain the procedure, we like to remark that proof systems based on propagation redundancy can easily simulate resolution: Since every resolvent of clauses in a formula *F* is implied by *F*, the assignment $$\alpha $$ blocked by the resolvent must falsify *F* and thus $$F{\,}|{\,}\alpha \vdash _{^{_{\!\!\!\raisebox {-5pt}{1}}}}\bot $$. We explain our procedure textually before we illustrate it in array notation:

First, we add the diagonal clauses $$D_1 = {\overline{x}}_{2,3} \vee {\overline{x}}_{4,1}$$ and $$D_2 = {\overline{x}}_{2,3} \vee {\overline{x}}_{4,2}$$ to $$ PHP _{3}$$. Now, we can derive the unit clause $${\overline{x}}_{2,3}$$ by resolving the two diagonal clauses $$D_1$$ and $$D_2$$ with the original pigeon hole clauses $$P_1 = {\overline{x}}_{2,3} \vee {\overline{x}}_{4,3}$$ and $$P_2 = x_{4,1} \vee x_{4,2} \vee x_{4,3}$$ as follows: We obtain $${\overline{x}}_{2,3} \vee x_{4,2} \vee x_{4,3}$$ by resolving $$D_1$$ with $$P_2$$. Then, we resolve this clause with $$D_2$$ to obtain $${\overline{x}}_{2,3} \vee x_{4,3}$$, which we resolve with $$P_1$$ to obtain $${\overline{x}}_{2,3}$$. Note that our proof system actually allows us to add $${\overline{x}}_{2,3}$$ immediately without carrying out all the resolution steps explicitly. Finally, we resolve $${\overline{x}}_{2,3}$$ with $$x_{2,1} \vee x_{2,2} \vee x_{2,3}$$ to obtain the desired clause $$x_{2,1} \vee x_{2,2}$$.

We next illustrate this procedure in array notation. We start by visualizing the clauses $D_1$, $D_2$, $P_1$, and $P_2$ that can be resolved to yield the clause ${\overline{x}}_{2,3}$. The clauses are given in array notation as follows: 
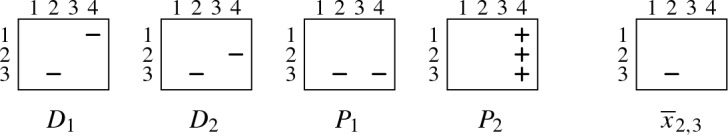


We can then resolve ${\overline{x}}_{2,3}$ with $x_{2,1} \vee x_{2,2} \vee x_{2,3}$ to obtain $x_{2,1} \vee x_{2,2}$: 
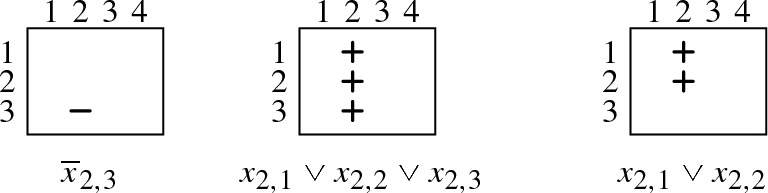


This should illustrate how a clause of the form $$x_{p,1} \vee \dots \vee x_{p,n}$$ ($$1 \le p \le n$$) can be reduced to a clause $$x_{p,1} \vee \dots \vee x_{p,n-1}$$. By repeating this procedure for every column *p* with $$1 \le p \le n$$, we can thus reduce a pigeon hole formula $$ PHP _{n}$$ to a pigeon hole formula $$ PHP _{n-1}$$ without introducing new variables. Note that the last step, in which we resolve the derived unit clause $${\overline{x}}_{2,3}$$ with the clause $$x_{2,1} \vee x_{2,2} \vee x_{2,3}$$, is actually not necessary for a valid $$\mathsf {PR}$$ proof of a pigeon hole formula, but we added it to simplify the presentation.

It remains to show that the diagonal clauses are indeed propagation redundant with respect to the pigeon hole formula. To do so, we show that for every assignment $\alpha = x_{p,n}x_{n+1,h}$ that is blocked by a diagonal clause ${\overline{x}}_{p,n} \vee {\overline{x}}_{n+1,h}$, it holds that for the assignment $\omega = {\overline{x}}_{p,n}{\overline{x}}_{n+1,h}x_{p,h}x_{n+1,n}$, $PHP _{n}{\,}|{\,}\alpha = PHP _{n}{\,}|{\,}\omega$, implying that $PHP _{n}{\,}|{\,}\alpha \vdash _{^{_{\!\!\!\raisebox {-5pt}{1}}}} PHP _{n}{\,}|{\,}\omega$. We also argue why other diagonal and unit clauses can be ignored when checking whether a new diagonal clause is propagation redundant.

We again illustrate the idea on $$ PHP _{3}$$. We now use array notation also for assignments, i.e., a “” (“”) in column *p* and row *h* denotes that the assignment makes variable $$x_{p,h}$$ true (false, respectively). Consider, for instance, the diagonal clause $$D_2 = {\overline{x}}_{2,3} \vee {\overline{x}}_{4,2}$$ that blocks $$\alpha = x_{2,3}x_{4,2}$$. The corresponding witnessing assignment $$\omega = {\overline{x}}_{2,3}{\overline{x}}_{4,2}x_{2,2}x_{4,3}$$ can be seen as a “rectangle” with two “” in the corners of one diagonal and two “” in the other corners: 
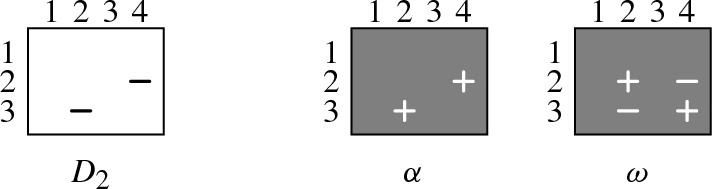
 To see that $$ PHP _{3}{\,}|{\,}\alpha $$ and $$ PHP _{3}{\,}|{\,}\omega $$ coincide on clauses $$x_{p,1} \vee \dots \vee x_{p,n}$$, consider that whenever $$\alpha $$ and $$\omega $$ assign a variable of such a clause, they both satisfy the clause (since they both have a “” in every column in which they assign a variable) and so they both remove it from $$ PHP _{3}$$. For instance, in the following example, both $$\alpha $$ and $$\omega $$ satisfy $$x_{2,1} \vee x_{2,2} \vee x_{2,3}$$ while both do not assign a variable of the clause $$x_{3,1} \vee x_{3,2} \vee x_{3,3}$$: 
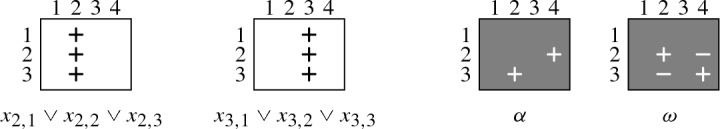
 To see that $PHP _{3}{\,}|{\,}\alpha$ and $PHP _{3}{\,}|{\,}\omega$ coincide on clauses of the form ${\overline{x}}_{p,h} \vee {\overline{x}}_{q,h}$, consider the following: If $\alpha$ falsifies a literal of ${\overline{x}}_{p,h} \vee {\overline{x}}_{q,h}$, then the resulting clause is a unit clause for which one of the two literals is not assigned by $\alpha$ (since $\alpha$ does not assign two variables in the same row). Now, one can show that the same unit clause is also contained in $PHP _{3}{\,}|{\,}\omega$, where it is obtained from another clause: Consider, for example, again the assignment $\alpha = x_{2,3}x_{4,2}$ and the corresponding witnessing assignment $\omega = {\overline{x}}_{2,3}{\overline{x}}_{4,2}x_{2,2}x_{4,3}$ from above. The assignment $\alpha$ turns the clause $C = {\overline{x}}_{3,2} \vee {\overline{x}}_{4,2}$ into the unit $C{\,}|{\,}\alpha = {\overline{x}}_{3,2}$. The same clause is contained in $PHP _{3}{\,}|{\,}\omega$, as it is obtained from $C' = {\overline{x}}_{2,2} \vee {\overline{x}}_{3,2}$ since $C'{\,}|{\,}\omega = C{\,}|{\,}\alpha = {\overline{x}}_{3,2}$: 
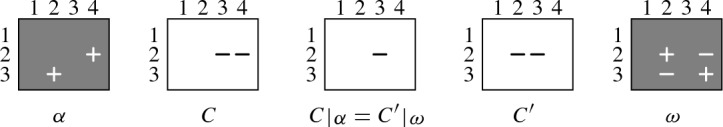
 Note that diagonal clauses and unit clauses that have been derived earlier can be ignored when checking whether the current one is propagation redundant. For instance, assume we are currently reducing $$ PHP _{n}$$ to $$ PHP _{n-1}$$. Then, the assignments $$\alpha $$ and $$\omega $$ under consideration only assign variables in $$ PHP _{n}$$. In contrast, the unit and diagonal clauses used for reducing $$ PHP _{n+1}$$ to $$ PHP _{n}$$ (or earlier ones) are only defined on variables outside of $$ PHP _{n}$$. They are therefore contained in both $$ PHP _{n}{\,}|{\,}\alpha $$ and $$ PHP _{n}{\,}|{\,}\omega $$. We can also ignore earlier unit and diagonal clauses over variables in $$ PHP _{n}$$, i.e., clauses used for reducing an earlier column or other diagonal clauses for the current column: If $$\alpha $$ assigns one of their variables, then $$\omega $$ satisfies them and so they are not in $$ PHP _{n}{\,}|{\,}\omega $$.

Finally, we want to mention that short $\mathsf {SPR}$ proofs (without new variables) of the pigeon hole formulas can be constructed by first adding $\mathsf {SPR}$ clauses of the form ${\overline{x}}_{p,n} \vee {\overline{x}}_{n+1,h} \vee x_{p,h} \vee x_{n+1,n}$ and then turning them into diagonal clauses using resolution. We left these proofs out since they are twice as large as the $\mathsf {PR}$ proofs and their explanation is less intuitive. A recent result shows that the conversion of a $\mathsf {PR}$ proof into a $\mathsf {DRAT}$ proof requires only one auxiliary variable [11]. We can thus construct short $\mathsf {DRAT}$ proofs without new variables from short $\mathsf {PR}$ proofs without new variables. To do so, we first eliminate a single variable from the original formula and then reuse that variable in the conversion algorithm. This is only possible because $\mathsf {DRAT}$ allows clause deletion. We consider it unlikely that there exist short proofs for the pigeon hole formulas in the $\mathsf {RAT}$/$\mathsf {LPR}$ proof system, where no deletions are allowed.

**Fig. 2:**
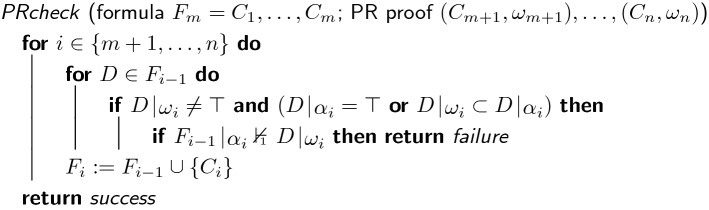
Pseudo code of the $\mathsf {PR}$-proof checking algorithm

## Evaluation

We implemented a $\mathsf {PR}$ proof checker on top of DRAT-trim [30]. The tool, formulas, and proofs are available at https://www.cs.utexas.edu/~marijn/pr. Figure 2 shows the pseudo code of the checking algorithm. The first “if” statement is not necessary but significantly improves the efficiency of the algorithm. The worst-case complexity of the algorithm is $\mathcal {O}(n^3)$, where *n* is the size of the final formula. The reason for this is that there are $n - m$ iterations of the outer for-loop and for each of these iterations, the inner for-loop is performed $|F_i|$ times, i.e., once for every clause in $F_i$. Given that $F_i$ contains *i* clauses, we know that the size of *F* is bounded by *n*. It follows that the inner for-loop is performed *mn* times. Now, there is a unit propagation test in the inner if-statement: If *k* is the maximal clause size and *n* is an upper bound for the size of the formula, then the complexity of unit propagation is known to be at most *kn*. Hence, the overall worst-case complexity of the algorithm is bounded by $m k n^2 = \mathcal {O}(n^3)$.

This complexity is the same as for $\mathsf {RAT}$-proof checking. In fact, the pseudo-code for $\mathsf {RAT}$-proof checking and $\mathsf {PR}$-proof checking is the same apart from the first if-statement, which is always true in the worst case, both for $\mathsf {RAT}$ and $\mathsf {PR}$. Although the theoretical worst-case complexity makes proof checking seem very expensive, it can be done quite efficiently in practice: For the $\mathsf {RAT}$ proofs produced by solvers in the SAT competitions, we observed that the runtime of proof checking is close to linear with respect to the sizes of the proofs.

Moreover, we want to highlight that verifying the $\mathsf {PR}$ property of a clause is relatively easy as long as a witnessing assignment is given. For an arbitrary clause *without* a witnessing assignment, however, it is an $\mathsf {NP}$-complete problem to decide if the clause is $\mathsf {PR}$ [15]. We therefore believe that in general, the verification of $\mathsf {PR}$ proofs is simpler than the actual solving/proving.

**Fig. 3:**
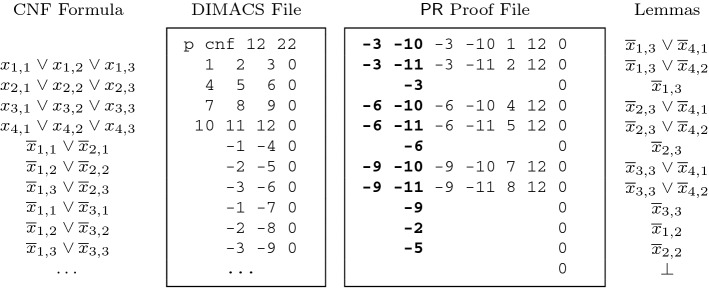
Left, ten clauses of $$ PHP _{3}$$ using the notation as elsewhere in this article and next to it the equivalent representation of these clauses in the DIMACS format used by SAT solvers. Right, the full $$\mathsf {PR}$$ refutation consisting of clause-witness pairs. A repetition of the first literal indicates the start of the optional witness

The format of $$\mathsf {PR}$$ proofs is an extension of $$\mathsf {DRAT}$$ proofs: the first numbers of line *i* denote the literals in $$C_i$$. Positive numbers refer to positive literals, and negative numbers refer to negative literals. In case a witness $$\omega _i$$ is provided, the first literal in the clause is repeated to denote the start of the witness. Recall that the witness always has to satisfy the clause. It is therefore guaranteed that the witness and the clause have at least one literal in common. Our format requires that such a literal occurs at the first position of the clause and of the witness. Finally, 0 marks the end of a line. Figure 3 shows the formula and the $$\mathsf {PR}$$ proof of our running example $$ PHP _{3}$$.

**Table 1:** The sizes (in terms of the number of variables and clauses) of pigeon hole formulas (top) and two-pigeons-per-hole formulas (bottom) as well as the sizes and validation times (in seconds) for their $\mathsf {PR}$ proofs (as described in Sect. 5) and their $\mathsf {DRAT}$ proofs (based on symmetry breaking [12])

Formula	Input		$\mathsf {PR}$ proofs			$\mathsf {DRAT}$ proofs		
	#var	#cls	#var	#cls	Time	#var	#cls	Time
hole10.cnf	110	561	110	385	0.17	440	3685	0.22
hole11.cnf	132	738	132	506	0.18	572	5236	0.23
hole12.cnf	156	949	156	650	0.19	728	7228	0.27
hole13.cnf	182	1197	182	819	0.21	910	9737	0.34
hole20.cnf	420	4221	420	2870	0.40	3080	49,420	2.90
hole30.cnf	930	13,981	930	9455	2.57	9920	234,205	61.83
hole40.cnf	1640	32,841	1640	22,140	13.54	22,960	715,040	623.29
hole50.cnf	2550	63,801	2550	42,925	71.72	44,200	1,708,925	3158.17
tph8.cnf	136	5457	136	680	0.32	3520	834,963	5.47
tph12.cnf	300	27,625	300	2300	1.81	11,376	28,183,301	1396.92
tph16.cnf	528	87,329	528	5456	11.16	Not available, too large		
tph20.cnf	820	213,241	820	10,660	61.69	Not available, too large		

Table 1 compares our $$\mathsf {PR}$$ proofs with existing $$\mathsf {DRAT}$$ proofs of the pigeon hole formulas and of formulas from another challenging benchmark suite of the SAT competition that allows two pigeons per hole. For the latter suite, $$\mathsf {PR}$$ proofs can be constructed in a similar way as those of the classical pigeon hole formulas. Notice that the $$\mathsf {PR}$$ proofs do not introduce new variables and that they contain fewer clauses than their corresponding formulas. The $$\mathsf {DRAT}$$ proof of $$ PHP _{n}$$ contains a copy of the formula $$ PHP _{k}$$ for each $$k < n$$. Checking $$\mathsf {PR}$$ proofs is also more efficient, as they are more compact.

## Properties of Propagation Redundancy

In the following, we discuss some properties of propagation redundancy and its restricted variants of literal-propagation redundancy and set-propagation redundancy. We first prove that if a clause *C* is either $\mathsf {LPR}$, $\mathsf {SPR}$, or $\mathsf {PR}$ with respect to a formula *F*, then every superclause of *C* (i.e., every clause *D* such that $C \subseteq D$) is also $\mathsf {LPR}$, $\mathsf {SPR}$, or $\mathsf {PR}$ (respectively) with respect to *F*. After this, we show how propagation-redundant clauses can be shortened based on unit propagation. Finally, we present an observation that clarifies the relationship between propagation-redundant clauses and their corresponding witnessing assignments.

Our strategy for showing that the superclauses of $\mathsf {LPR}$, $\mathsf {SPR}$, and $\mathsf {PR}$ clauses are also $\mathsf {LPR}$, $\mathsf {SPR}$, and $\mathsf {PR}$ is as follows: Assume, for instance, that *C* is a $\mathsf {PR}$ clause with respect to *F* and let $\alpha$ be the assignment blocked by *C*. We then know that there exists an assignment $\omega$ such that $F{\,}|{\,}\alpha \vdash _{^{_{\!\!\!\raisebox {-5pt}{1}}}}F{\,}|{\,}\omega$. To show that every superclause $C'$ of *C* is also propagation redundant with respect to *F*, we extend $\alpha$ so that it becomes the assignment $\alpha '$ blocked by $C'$. After this, we extend $\omega$ to an assignment $\omega '$ such that $F{\,}|{\,}\alpha ' \vdash _{^{_{\!\!\!\raisebox {-5pt}{1}}}}F{\,}|{\,}\omega '$. The following example shows that we cannot simply extend $\alpha$ without extending $\omega$:

### Example 5

Let $F = (x \vee y) \wedge ({\overline{x}} \vee y)$ and let $\alpha = x$ and $\omega = {\overline{x}}$. Then, $F{\,}|{\,}\alpha$ and $F{\,}|{\,}\omega$ both contain only the unit clause *y* and so it holds that $F{\,}|{\,}\alpha \vdash _{^{_{\!\!\!\raisebox {-5pt}{1}}}}F{\,}|{\,}\omega$. If we only extend $\alpha$ to $\alpha y$, then $F{\,}|{\,}\alpha y$ contains no clauses and thus $F{\,}|{\,}\alpha y \nvdash _{^{_{\!\!\!\raisebox {-5pt}{1}}}}F{\,}|{\,}\omega$. However, if we also extend $\omega$ to $\omega y$, then we again have $F{\,}|{\,}\alpha y \vdash _{^{_{\!\!\!\raisebox {-5pt}{1}}}}F{\,}|{\,}\omega y$. $\square$

In the following, we present several statements that help us extend $\omega$ in the right way. We start with a simple observation about the relation between unit propagation and variable assignments. If a formula is unsatisfiable, then the formula remains unsatisfiable after we assign some of its variables. A similar property holds when unsatisfiability can be shown by unit propagation: If unit propagation derives a conflict on a formula, then we can assign truth values to arbitrary variables of the formula and unit propagation will still derive a conflict. This property will be useful below when we prove other properties about propagation redundancy:

### Proposition 5

If unit propagation derives a conflict on a formula *F*, then it derives a conflict on $F{\,}|{\,}x$ for every literal *x*.

### Proof

Assume unit propagation derives a conflict on *F*. Then, there must exist a sequence $$C_1, \dots , C_k$$ of clauses from *F* such that $$C_1$$ is the unit clause $$a_1$$, $$C_2{\,}|{\,}a_1$$ is the unit clause $$a_2$$, and so on until $$C_{k-1}{\,}|{\,}a_1\dots a_{n-2} = a_{k-1}$$, and finally $$C_{k}{\,}|{\,}a_1\dots a_{k-1} = \bot $$. Now, if the variable $$ var (x)$$ does not occur in any of the clauses $$C_1,\dots ,C_k$$, then $$C_i{\,}|{\,}x = C_i$$ for each $$i \in 1,\dots , n$$ and thus unit propagation derives a conflict on $$F{\,}|{\,}x$$. Assume now that $$ var (x)$$ occurs in $$C_1, \dots , C_k$$ and let $$C_i$$ be the clause with the smallest *i* such that $$ var (x) \in C_i$$. Then, $$C_1, \dots , C_{i-1} \in F{\,}|{\,}x$$ and so unit propagation derives the unit clauses $$a_1, \dots , a_{i-1}$$ on $$F{\,}|{\,}x$$. We proceed by a case distinction.

$$x \in C_i$$: In this case, $$C_i \notin F{\,}|{\,}x$$, but we know that $$C_i{\,}|{\,}a_1\dots a_{i-1} = a_i = x$$ since $$ var (x)$$ cannot occur in $$a_1, \dots , a_{i-1}$$. But then the assignment $$a_1 \dots a_{i-1} a_i$$, derived by unit propagation on *F*, is the assignment $$a_1 \dots a_{i-1} x$$, derived by unit propagation on $$F{\,}|{\,}x$$. Hence, unit propagation on $$F{\,}|{\,}x$$ derives a conflict using the clauses $$C_1{\,}|{\,}x, \dots , C_{i-1}{\,}|{\,}x, C_{i+1}{\,}|{\,}x, \dots C_k{\,}|{\,}x$$.

${\overline{x}} \in C_i$: In that case, $C_i{\,}|{\,}a_1\dots a_{i-1}$ must be the unit clause ${\overline{x}}$. It follows that $C_i \subseteq {\overline{a}}_1 \vee \dots \vee {\overline{a}}_{i-1} \vee {\overline{x}}$ and thus $C_i{\,}|{\,}x \subseteq {\overline{a}}_1 \vee \dots \vee {\overline{a}}_{i-1}$. Hence, unit propagation on $F{\,}|{\,}x$ derives a conflict with the clauses $C_1, C_2, \dots , C_i{\,}|{\,}x$ since it derives all the unit clauses $a_1, \dots , a_{i-1}$. $\square$

In Example 5, we presented a formula *F* with two assignments $\alpha$ and $\omega$ such that $F{\,}|{\,}\alpha \vdash _{^{_{\!\!\!\raisebox {-5pt}{1}}}}F{\,}|{\,}\omega$. After extending $\alpha$ to $\alpha x$, however, we could observe that $F{\,}|{\,}\alpha x \nvdash _{^{_{\!\!\!\raisebox {-5pt}{1}}}}F{\,}|{\,}\omega$. The problem in the example is that in contrast to $F{\,}|{\,}\alpha$, the formula $F{\,}|{\,}\alpha x$ does not contain the unit clause *x* anymore while $F{\,}|{\,}\omega$ still contains *x* as a clause. Hence, $F{\,}|{\,}\alpha x$ does not imply $F{\,}|{\,}\omega$. However, as the next statement tells us, it is guaranteed that $F{\,}|{\,}\alpha x$ implies all those clauses of $F{\,}|{\,}\omega$ that contain neither *x* nor ${\overline{x}}$.

In the rest of this section, given a clause $C = (c_1 \vee \dots \vee c_n)$, we write $\lnot C$ for the conjunction ${\overline{c}}_1 \wedge \dots \wedge {\overline{c}}_n$ of unit clauses. In the following statement, the requirement that $\alpha$ must not falsify *x* makes sure that $\alpha x$ is well-defined:

### Lemma 6

Let *F* be formula, let $\alpha , \omega$ be assignments such that $F{\,}|{\,}\alpha \vdash _{^{_{\!\!\!\raisebox {-5pt}{1}}}}F{\,}|{\,}\omega$, and let *x* be a literal that is not falsified by $\alpha$. Then, $F{\,}|{\,}\alpha x \vdash _{^{_{\!\!\!\raisebox {-5pt}{1}}}}D{\,}|{\,}\omega$ for every clause $D{\,}|{\,}\omega \in F{\,}|{\,}\omega$ such that $var (x) \notin var (D{\,}|{\,}\omega )$.

### Proof

Assume that $F{\,}|{\,}\alpha \vdash _{^{_{\!\!\!\raisebox {-5pt}{1}}}}F{\,}|{\,}\omega$ and let $D{\,}|{\,}\omega \in F{\,}|{\,}\omega$ be a clause such that $var (x) \notin D{\,}|{\,}\omega$. Since $F{\,}|{\,}\alpha \vdash _{^{_{\!\!\!\raisebox {-5pt}{1}}}}D{\,}|{\,}\omega$, we know that unit propagation derives a conflict on $F{\,}|{\,}\alpha \wedge \lnot (D{\,}|{\,}\omega )$, with $\lnot (D{\,}|{\,}\omega )$ being the conjunction of the negated literals of $D{\,}|{\,}\omega$. Since $var (x) \notin var (D{\,}|{\,}\omega )$, it follows that $D{\,}|{\,}\omega = D{\,}|{\,}\omega x$. Thus, $(F{\,}|{\,}\alpha \wedge \lnot (D{\,}|{\,}\omega )){\,}|{\,}x = F{\,}|{\,}\alpha x \wedge \lnot (D{\,}|{\,}\omega )$. But then, since unit propagation derives a conflict on $F{\,}|{\,}\alpha \wedge \lnot (D{\,}|{\,}\omega )$, we know, by Proposition 5, that unit propagation derives a conflict on $F{\,}|{\,}\alpha x \wedge \lnot (D{\,}|{\,}\omega )$. It follows that $F{\,}|{\,}\alpha x \vdash _{^{_{\!\!\!\raisebox {-5pt}{1}}}}D{\,}|{\,}\omega$. $\square$

Using Lemma 6, we can show that every literal that is neither falsified by $\alpha$ nor by $\omega$ can just be appended to both $\alpha$ and $\omega$:

### Lemma 7

Let *F* be formula, $\alpha$ and $\omega$ assignments, and *x* a literal that is neither falsified by $\alpha$ nor by $\omega$. Then, $F{\,}|{\,}\alpha \vdash _{^{_{\!\!\!\raisebox {-5pt}{1}}}}F{\,}|{\,}\omega$ implies $F{\,}|{\,}\alpha x \vdash _{^{_{\!\!\!\raisebox {-5pt}{1}}}}F{\,}|{\,}\omega x$.

### Proof

Suppose $F{\,}|{\,}\alpha \vdash _{^{_{\!\!\!\raisebox {-5pt}{1}}}}F{\,}|{\,}\omega$ and let $D{\,}|{\,}\omega x \in F{\,}|{\,}\omega x$ for $D \in F$. We show that $F{\,}|{\,}\alpha x \vdash _{^{_{\!\!\!\raisebox {-5pt}{1}}}}D{\,}|{\,}\omega x$. If ${\overline{x}} \in D{\,}|{\,}\omega$, then $D{\,}|{\,}\omega = D{\,}|{\,}\omega x \vee {\overline{x}}$. Hence, unit propagation derives a conflict on $F{\,}|{\,}\alpha \wedge \lnot (D{\,}|{\,}\omega x) \wedge x$, with $\lnot (D{\,}|{\,}\omega x)$ being the conjunction of the negated literals of $D{\,}|{\,}\omega x$. But then unit propagation derives a conflict on $F{\,}|{\,}\alpha x \wedge \lnot (D{\,}|{\,}\omega x)$ and thus $F{\,}|{\,}\alpha x \vdash _{^{_{\!\!\!\raisebox {-5pt}{1}}}}D{\,}|{\,}\omega x$. If ${\overline{x}} \notin D$, then $D{\,}|{\,}\omega x = D{\,}|{\,}\omega$. Now, we know that $F{\,}|{\,}\alpha \vdash _{^{_{\!\!\!\raisebox {-5pt}{1}}}}D{\,}|{\,}\omega$ and hence $F{\,}|{\,}\alpha \vdash _{^{_{\!\!\!\raisebox {-5pt}{1}}}}D{\,}|{\,}\omega x$. Thus, by Lemma 6, it follows that $F{\,}|{\,}\alpha x \vdash _{^{_{\!\!\!\raisebox {-5pt}{1}}}}D{\,}|{\,}\omega x$. $\square$

Assume that a clause *C* is $\mathsf {LPR}$ with respect to a formula *F*. Let *D* be a superclause of *C*, $\alpha$ be the assignment blocked by *C*, and $\alpha x_1 \dots x_k$ the assignment blocked by *D*. Then, we know that there exists a literal $l \in C$ such that $F{\,}|{\,}\alpha \vdash _{^{_{\!\!\!\raisebox {-5pt}{1}}}}F{\,}|{\,}\alpha _l$. But then Lemma 7 tells us that $F{\,}|{\,}\alpha x_1 \dots x_k \vdash _{^{_{\!\!\!\raisebox {-5pt}{1}}}}F{\,}|{\,}\alpha _l x_1 \dots x_k$ and thus *D* is $\mathsf {LPR}$ with respect to *F*. The same argument applies to $\mathsf {SPR}$ clauses (but not to $\mathsf {PR}$ clauses in general) and thus we get:

### Theorem 8

If a clause *C* is $\mathsf {LPR}$ ($\mathsf {SPR}$) with respect to *F*, then every superclause of *C* is $\mathsf {LPR}$ ($\mathsf {SPR}$, respectively) with respect to *F*.

To show that the corresponding statement also holds for $\mathsf {PR}$ clauses that are not $\mathsf {SPR}$ clauses, we need to show some additional properties of $\mathsf {PR}$ clauses. The next statement, which is a simple consequence of Lemma 6, tells us that the extension of $\alpha$ to $\alpha x$ is harmless if $\omega$ already falsifies *x*:

### Lemma 9

Let *F* be formula, let $\alpha$ and $\omega$ be assignments, and let *x* be a literal that is not falsified by $\alpha$. Then, $F{\,}|{\,}\alpha \vdash _{^{_{\!\!\!\raisebox {-5pt}{1}}}}F{\,}|{\,}\omega {\overline{x}}$ implies $F{\,}|{\,}\alpha x \vdash _{^{_{\!\!\!\raisebox {-5pt}{1}}}}F{\,}|{\,}\omega {\overline{x}}$.

### Proof

Assume that $$ var (x)$$ occurs in a clause $$D \in F$$. If $$x \in D$$, then $$D{\,}|{\,}\omega {\overline{x}}$$ does not contain *x*. If $${\overline{x}} \in D$$, then *D* is satisfied by $$\omega {\overline{x}}$$ and thus $$D{\,}|{\,}\omega {\overline{x}} \notin F{\,}|{\,}\omega {\overline{x}}$$. Thus, by Lemma 6, $$F{\,}|{\,}\alpha x \vdash _{^{_{\!\!\!\raisebox {-5pt}{1}}}}F{\,}|{\,}\omega {\overline{x}}$$. $$\square $$

Putting everything together, we can now show that we can always extend $\alpha$ if we just extend $\omega$ accordingly:

### Lemma 10

Let *F* be a formula, let $\alpha$ and $\omega$ be two assignments such that $F{\,}|{\,}\alpha \vdash _{^{_{\!\!\!\raisebox {-5pt}{1}}}}F{\,}|{\,}\omega$, and let $\alpha '$ be an assignment such that $\alpha \subseteq \alpha '$. Then, there exists an assignment $\omega '$ such that $\omega \subseteq \omega '$ and $F{\,}|{\,}\alpha ' \vdash _{^{_{\!\!\!\raisebox {-5pt}{1}}}}F{\,}|{\,}\omega '$.

### Proof

Suppose $\alpha '$ is an assignment such that $\alpha \subseteq \alpha '$. Then, $\alpha '$ is of the form $\alpha x_1 \dots x_k$ where $n \in 0, \dots , n$. Now, starting with $x_1$, we stepwise extend $\alpha$ with $x_1, \dots , x_k$ to finally obtain $\alpha x_1 \dots x_k$. We just have to extend $\omega$ to an assignment $\omega _k$ accordingly to ensure that $F{\,}|{\,}\alpha x_1 \dots x_k \vdash _{^{_{\!\!\!\raisebox {-5pt}{1}}}}F{\,}|{\,}\omega _k$. We start with $\omega _0 = \omega$ and proceed as follows for $i \in 1, \dots , n$: If $var (x_i) \in var (\omega )$, define $\omega _i = \omega _{i-1}$. In contrast, if $var (x_i) \notin var (\omega )$, define $\omega _i = \omega _{i-1} x_i$.

By a simple induction on *i* we can now show $F{\,}|{\,}\alpha x_1 \dots x_i \vdash _{^{_{\!\!\!\raisebox {-5pt}{1}}}}F{\,}|{\,}\omega _i$ for every $i \in 0, \dots , k$: The base case, $F{\,}|{\,}\alpha \vdash _{^{_{\!\!\!\raisebox {-5pt}{1}}}}F{\,}|{\,}\omega$, holds by assumption. The induction hypothesis states that $F{\,}|{\,}\alpha x_1 \dots x_{i-1} \vdash _{^{_{\!\!\!\raisebox {-5pt}{1}}}}F{\,}|{\,}\omega _{i-1}$. Now, for the induction step, if $\omega (x_i) = 0$, then $\omega _{i}$ is of the form $\omega _{i-1}{\overline{x}}_i$. In this case, by Lemma 9, $F{\,}|{\,}\alpha x_1 \dots x_i \vdash _{^{_{\!\!\!\raisebox {-5pt}{1}}}}F{\,}|{\,}\omega _{i-1} {\overline{x}}_i$. If $var (x_i) \notin var (\omega )$ or $\omega (x_i) = 1$, then $\omega _i$ is of the form $\omega _{i-1} x_i$. In that case, by Lemma 7, $F{\,}|{\,}\alpha x_1 \dots x_i \vdash _{^{_{\!\!\!\raisebox {-5pt}{1}}}}F{\,}|{\,}\omega _{i-1} x_i$ . $\square$

As immediate consequence we obtain one of our main statements:

### Theorem 11

If a clause *C* is $\mathsf {PR}$ with respect to *F*, then every superclause of *C* is $\mathsf {PR}$ with respect to *F*.

Next, we show that we can remove certain literals from propagation-redundant clauses without violating their property of being propagation redundant. The idea is as follows: Let *C* be a clause that is propagation redundant with respect to a formula *F* and let $\alpha x_1 \dots x_k$ be the assignment blocked by *C*. Now, if unit propagation on $F{\,}|{\,}\alpha$ derives the unit clauses $x_1, \dots , x_k$, then the clause that blocks only $\alpha$—and not the whole assignment $\alpha x_1 \dots x_k$—is propagation redundant with respect to *F* too. To show this, we first introduce the notion of *propagation extensions*. Note that in the following definition, by a *consistent* set of unit clauses, we mean a set of unit clauses that does not contain two complementary unit clauses *x* and ${\overline{x}}$:

### Definition 8

Let *F* be a formula, let $\alpha$ be an assignment, and let $\{x_1, \dots , x_k\}$ be a consistent set of unit clauses derived by unit propagation on $F{\,}|{\,}\alpha$. Then, $\alpha x_1 \dots x_k$ is a *propagation extension* of $\alpha$ on *F*.

### Example 6

Let $F = (x \vee y) \wedge ({\overline{y}} \vee z)$ and let $\alpha = {\overline{x}}$. Unit propagation on $F{\,}|{\,}\alpha$ derives the unit clauses *y* and *z*. Hence, the assignments $\alpha y$, $\alpha z$, and $\alpha y z$ are propagation extensions of $\alpha$ on *F*. Now consider the formula $F \wedge {\overline{z}}$. Then, unit propagation on $(F \wedge {\overline{z}}){\,}|{\,}\alpha$ derives the unit clauses *y*, *z*, and ${\overline{z}}$. Thus, the propagation extensions of $\alpha$ on $F \wedge ({\overline{z}})$ are the assignments $\alpha {\overline{z}}$ and $\alpha y {\overline{z}}$ as well as all the propagation extensions of $\alpha$ on *F*. $\square$

If $F{\,}|{\,}\alpha \!^{+} \vdash _{^{_{\!\!\!\raisebox {-5pt}{1}}}}F{\,}|{\,}\omega$ for some propagation extension $\alpha \!^{+}$ of an assignment $\alpha$, we can simply shorten $\alpha \!^{+}$ to $\alpha$ without modifying $\omega$ and it will still hold that $F{\,}|{\,}\alpha \vdash _{^{_{\!\!\!\raisebox {-5pt}{1}}}}F{\,}|{\,}\omega$:

### Lemma 12

Let *F* be a formula, $\alpha$ an assignment, and $\alpha \!^{+}$ a propagation extension of $\alpha$ on *F*. Then, $F{\,}|{\,}\alpha \vdash _{^{_{\!\!\!\raisebox {-5pt}{1}}}}F{\,}|{\,}\omega$ if and only if $F{\,}|{\,}\alpha \!^{+} \vdash _{^{_{\!\!\!\raisebox {-5pt}{1}}}}F{\,}|{\,}\omega$.

### Proof

The “only if” direction is an immediate consequence of Lemma 10. For the “if” direction, assume that $F{\,}|{\,}\alpha \!^{+} \vdash _{^{_{\!\!\!\raisebox {-5pt}{1}}}}F{\,}|{\,}\omega$ and let $D{\,}|{\,}\omega \in F{\,}|{\,}\omega$. We know that unit propagation derives a conflict on $F{\,}|{\,}\alpha \!^{+} \wedge \lnot (D{\,}|{\,}\omega )$ where $\lnot (D{\,}|{\,}\omega )$ is the conjunction of the negated literals of $D{\,}|{\,}\omega$. Since unit propagation derives $F{\,}|{\,}\alpha \!^{+}$ from $F{\,}|{\,}\alpha$, it follows that unit propagation derives a conflict on $F{\,}|{\,}\alpha \wedge \lnot (D{\,}|{\,}\omega )$. Hence, $F{\,}|{\,}\alpha \vdash _{^{_{\!\!\!\raisebox {-5pt}{1}}}}D{\,}|{\,}\omega$ and thus $F{\,}|{\,}\alpha \vdash _{^{_{\!\!\!\raisebox {-5pt}{1}}}}F{\,}|{\,}\omega$. $\square$

Using Lemma 12, we can now show that the removal of propagated literals from $\mathsf {PR}$ clauses is harmless:

### Theorem 13

Let *C* be a clause that is $\mathsf {PR}$ with respect to a formula *F* and let $\alpha \!^{+}$ be the assignment blocked by *C*. If $\alpha \!^{+}$ is a propagation extension of an assignment $\alpha$ on *F*, then the clause that blocks $\alpha$ is $\mathsf {PR}$ with respect to *F*.

### Proof

Assume that $\alpha \!^{+}$ is a propagation extension of an assignment $\alpha$ on *F* and let $C^-$ be the clause that blocks $\alpha$. Then, $\alpha \!^{+}$ is of the form $\alpha x_1 \dots x_k$ where $x_1, \dots x_k$ are all the literals derived by unit propagation on $F{\,}|{\,}\alpha$. Since *C* is propagation redundant with respect to *F*, we know that there exists some assignment $\omega$ such that $\omega$ satisfies *C* and $F{\,}|{\,}\alpha x_1 \dots x_k \vdash _{^{_{\!\!\!\raisebox {-5pt}{1}}}}F{\,}|{\,}\omega$. Hence, by Lemma 12, $F{\,}|{\,}\alpha \vdash _{^{_{\!\!\!\raisebox {-5pt}{1}}}}F{\,}|{\,}\omega$. Now, if $\omega$ satisfies $C^-$, then $C^-$ is propagation redundant with respect to *F*.

Assume thus that $\omega$ does not satisfy $C^-$. Then, since $\omega$ satisfies *C*, it must falsify a literal in $x_1, \dots , x_k$. Let $x_i$ be the first literal (i.e., the one with the smallest index) of $x_1, \dots , x_k$ that is falsified by $\omega$. Then, there exists a clause $D \in F$ such that $D{\,}|{\,}\alpha x_1 \dots x_{i-1}$ is the unit clause $x_i$ and thus $\omega$ falsifies *D*. Hence, $F{\,}|{\,}\alpha \vdash _{^{_{\!\!\!\raisebox {-5pt}{1}}}}\bot$. But then, $C^-$ is trivially $\mathsf {PR}$ with respect to *F* since it holds for every assignment $\tau$ (and in particular for every $\tau$ that satisfies *C*) that $F{\,}|{\,}\alpha \vdash _{^{_{\!\!\!\raisebox {-5pt}{1}}}}F{\,}|{\,}\tau$. $\square$

We can prove a corresponding result about $\mathsf {LPR}$ clauses: If we remove propagated literals from an $\mathsf {LPR}$ clause, then the resulting clause is also an $\mathsf {LPR}$ clause. As we will see later, this is not the case for $\mathsf {SPR}$ clauses. We start with two lemmas:

### Lemma 14

Let *F* be a formula, $\alpha$ and $\omega$ assignments, and *x* a literal such that $F{\,}|{\,}\alpha \vdash _{^{_{\!\!\!\raisebox {-5pt}{1}}}}x$. Then, $F{\,}|{\,}\alpha \vdash _{^{_{\!\!\!\raisebox {-5pt}{1}}}}F{\,}|{\,}\omega x$ implies $F{\,}|{\,}\alpha \vdash _{^{_{\!\!\!\raisebox {-5pt}{1}}}}F{\,}|{\,}\omega$.

### Proof

Suppose to the contrary that there exists a clause $D{\,}|{\,}\omega \in F{\,}|{\,}\omega$ such that $F{\,}|{\,}\alpha \nvdash _{^{_{\!\!\!\raisebox {-5pt}{1}}}}D{\,}|{\,}\omega$. Then, *D* must contain *x*, for otherwise $\omega x$ would not satisfy *D*, which would in turn imply $F{\,}|{\,}\alpha \nvdash _{^{_{\!\!\!\raisebox {-5pt}{1}}}}F{\,}|{\,}\omega x$. Therefore, the clause $\lnot (D{\,}|{\,}\omega )$, being the conjunction of the negated literals of $D{\,}|{\,}\omega$, must contain ${\overline{x}}$. But then, since unit propagation on $F{\,}|{\,}\alpha$ derives *x*, it follows that unit propagation derives a conflict on $F{\,}|{\,}\alpha \wedge \lnot (D{\,}|{\,}\omega )$. Hence, $F{\,}|{\,}\alpha \vdash _{^{_{\!\!\!\raisebox {-5pt}{1}}}}D{\,}|{\,}\omega$ and thus $F{\,}|{\,}\alpha \vdash _{^{_{\!\!\!\raisebox {-5pt}{1}}}}F{\,}|{\,}\omega$. $\square$

### Lemma 15

Let *F* be a formula, $\alpha$ an assignment, $\alpha \!^{+}$ a propagation extension of $\alpha$ on *F*, and *l* a literal. Then, $F{\,}|{\,}\alpha \!^{+} \vdash _{^{_{\!\!\!\raisebox {-5pt}{1}}}}F{\,}|{\,}\alpha _l\!\!^{+}$ implies $F{\,}|{\,}\alpha \vdash _{^{_{\!\!\!\raisebox {-5pt}{1}}}}F{\,}|{\,}\alpha _l$.

### Proof

Assume that $\alpha \!^{+}$ is a propagation extension of $\alpha$. Then, $\alpha \!^{+}$ is of the form $\alpha \tau$ where $\tau$ make the literals true that have been derived by unit propagation on $F{\,}|{\,}\alpha$. To show that $F{\,}|{\,}\alpha \vdash _{^{_{\!\!\!\raisebox {-5pt}{1}}}}F{\,}|{\,}\alpha _l$, we distinguish two cases:

$var (l) \in var (\alpha )$: In this case, $F{\,}|{\,}\alpha \tau \vdash _{^{_{\!\!\!\raisebox {-5pt}{1}}}}F{\,}|{\,}\alpha _l \tau$ and thus $F{\,}|{\,}\alpha \vdash _{^{_{\!\!\!\raisebox {-5pt}{1}}}}F{\,}|{\,}\alpha _l \tau$. Now, since $F{\,}|{\,}\alpha \vdash _{^{_{\!\!\!\raisebox {-5pt}{1}}}}x$ for every literal *x* satisfied by $\tau$, we use Lemma 14 to repeatedly remove from $\alpha _l \tau$ all assignments made by $\tau$ to obtain $F{\,}|{\,}\alpha \vdash _{^{_{\!\!\!\raisebox {-5pt}{1}}}}F{\,}|{\,}\alpha _l$.

$var (l) \in var (\tau )$: In that case, $F{\,}|{\,}\alpha \tau \vdash _{^{_{\!\!\!\raisebox {-5pt}{1}}}}F{\,}|{\,}\alpha \tau _l$. Since unit propagation on $F{\,}|{\,}\alpha$ derives ${\overline{l}}$, there exists a clause $D \in F$ such that $\alpha \tau _l$—which satisfies *l*—falsifies *D*. Hence, $D{\,}|{\,}\alpha \tau _l = \bot$, and since $F{\,}|{\,}\alpha \tau \vdash _{^{_{\!\!\!\raisebox {-5pt}{1}}}}D{\,}|{\,}\alpha \tau _l$, it follows that unit propagation derives a conflict on $F {\,}|{\,}\alpha \tau$. But then unit propagation must derive a conflict on $F{\,}|{\,}\alpha$ and thus $F{\,}|{\,}\alpha$ implies every clause via unit propagation. We thus conclude that $F{\,}|{\,}\alpha \vdash _{^{_{\!\!\!\raisebox {-5pt}{1}}}}F{\,}|{\,}\alpha _l$. $\square$

The following Theorem is now an immediate consequence of Lemma 15:

### Theorem 16

Let *C* be a clause that is $\mathsf {LPR}$ with respect to a formula *F* and let $\alpha \!^{+}$ be the assignment blocked by *C*. If $\alpha \!^{+}$ is a propagation extension of an assignment $\alpha$ on *F*, then the clause that blocks $\alpha$ is $\mathsf {LPR}$ with respect to *F*.

The corresponding property does not hold for $\mathsf {SPR}$ clauses, as illustrated by the following example:

### Example 7

Let $F = ({\overline{x}} \vee y) \wedge (x \vee {\overline{y}})$, $C = x \vee y$, and $L = \{x, y\}$. Then, $\alpha \!^{+} = {\overline{x}}\,{\overline{y}}$, which is the assignment blocked by *C*, is a propagation extension of the assignment $\alpha = {\overline{x}}$. Moreover, since $F{\,}|{\,}\alpha _L\!\!\!\!\!^{+}$ contains no clauses, we have $F{\,}|{\,}\alpha \!^{+} \vdash _{^{_{\!\!\!\raisebox {-5pt}{1}}}}F{\,}|{\,}\alpha _L\!\!\!\!\!^{+}$ and thus *C* is set-propagation redundant with respect to *F*. However, $F{\,}|{\,}\alpha \nvdash _{^{_{\!\!\!\raisebox {-5pt}{1}}}}F{\,}|{\,}\alpha _x$ and thus the subclause *x* of *C* is not set-propagation redundant with respect to *F*. $\square$

We conclude this section with an observation about the witnessing assignments of propagation-redundant clauses. In the case of propagation redundancy, the domain of the witnessing assignment $\omega$ is not constrained to a particular set of variables. Thus, if we are given a clause *C* and we want to find a corresponding assignment $\omega$ that witnesses the propagation redundancy of *C*, we would have to consider assignments over all possible sets of variables. It turns out, however, that there is always a witnessing assignment that assigns all variables occurring in *C*, and possibly more. Thus, if $\alpha$ is the assignment blocked by *C*, we only need to consider assignments $\omega$ such that $var (\alpha ) \subseteq var (\omega )$. The reason for this is that we can extend every witness $\omega$ to the variables of $var (\alpha )$:

### Proposition 17

Let *F* be a formula, $\alpha$ and $\omega$ assignments, and *x* a literal such that $var (x) \in var (\alpha ) \setminus var (\omega )$. Then, $F{\,}|{\,}\alpha \vdash _{^{_{\!\!\!\raisebox {-5pt}{1}}}}F{\,}|{\,}\omega$ implies $F{\,}|{\,}\alpha \vdash _{^{_{\!\!\!\raisebox {-5pt}{1}}}}F {\,}|{\,}\omega x$.

### Proof

Let $D{\,}|{\,}\omega x \in F{\,}|{\,}\omega x$. We show that $F{\,}|{\,}\alpha \vdash _{^{_{\!\!\!\raisebox {-5pt}{1}}}}D{\,}|{\,}\omega x$. Clearly, *x* is not contained in *D* for otherwise $D{\,}|{\,}\omega x = \top$. Therefore, the only possible difference between $D{\,}|{\,}\omega$ and $D{\,}|{\,}\omega x$ is that ${\overline{x}}$ is contained in $D{\,}|{\,}\omega$ but not in $D{\,}|{\,}\omega x$. Now, since $var (x) \in var (\alpha )$, we know that $var (x) \notin F{\,}|{\,}\alpha$. But then, $F{\,}|{\,}\alpha \vdash _{^{_{\!\!\!\raisebox {-5pt}{1}}}}D{\,}|{\,}\omega x$ if and only if $F{\,}|{\,}\alpha \vdash _{^{_{\!\!\!\raisebox {-5pt}{1}}}}D{\,}|{\,}\omega$. It thus follows that $F{\,}|{\,}\alpha \vdash _{^{_{\!\!\!\raisebox {-5pt}{1}}}}F {\,}|{\,}\omega x$. $\square$

## Related Work

Here, we discuss how the concepts in this article are related to *variable instantiation* [1], *autarkies* [24], *safe assignments* [29], and *symmetry breaking* [6]. If $F{\,}|{\,}{\overline{x}} \vDash F{\,}|{\,}x$ holds for some literal *x*, then *variable instantiation*, as described by Andersson et al. [1], allows to make the literal *x* true in the formula *F*. Analogously, our redundancy notion identifies the clause *x* as redundant.

As presented by Monien and Speckenmeyer [24], an assignment $\omega$ is an *autarky* for a formula *F* if it satisfies all clauses of *F* that contain a literal to which $\omega$ assigns a truth value. If an assignment $\omega$ is an autarky for a formula *F*, then *F* is satisfiability equivalent to $F{\,}|{\,}\omega$. Similarly, propagation redundancy $\mathsf {PR}$ allows us to add all the unit clauses satisfied by an autarky, with the autarky serving as a witness:^2^ Let $\omega$ be an autarky for some formula *F*, $C = x$ for a literal *x* satisfied by $\omega$, and $\alpha = {\overline{x}}$ the assignment blocked by *C*. Notice that $F{\,}|{\,}\alpha \supseteq F{\,}|{\,}\omega$ and thus *C* is $\mathsf {PR}$ with respect to *F*.

According to Weaver and Franco [29], an assignment $\omega$ is considered *safe* if, for every assignment $\alpha$ with $var (\alpha ) = var (\omega )$, it holds that $F{\,}|{\,}\alpha \vDash F{\,}|{\,}\omega$. If an assignment $\omega$ is safe, then $F{\,}|{\,}\omega$ is satisfiability equivalent to *F*. In a similar fashion, our approach allows us to block all the above-mentioned assignments $\alpha \ne \omega$. Through this, we obtain a formula that is logically equivalent to $F{\,}|{\,}\omega$. Note that safe assignments generalize autarkies and variable instantiation. Moreover, while safe assignments only allow the application of an assignment $\omega$ to a formula *F* if $F{\,}|{\,}\alpha \vDash F{\,}|{\,}\omega$ holds for *all* assignments $\alpha \ne \omega$, our approach enables us to block an assignment $\alpha$ as soon as $F{\,}|{\,}\alpha \vDash F{\,}|{\,}\omega$.

Finally, symmetry breaking [6] can be expressed in the $\mathsf {DRAT}$ proof system [12] but existing methods introduce many new variables and duplicate the input formula multiple times. It might be possible to express symmetry breaking without new variables in the $\mathsf {PR}$ proof system. For one important symmetry, row-interchangeability [7], the symmetry breaking using $\mathsf {PR}$ without new variables appears similar to the method we presented for the pigeon hole formulas.

## Open Problems and Future Work

In a more recent paper, we showed that there exists a polynomial-time procedure that translates $\mathsf {PR}$ proofs to $\mathsf {DRAT}$ proofs by introducing one new variable [11]. Moreover, we proved that extended resolution polynomially simulates the $\mathsf {DRAT}$ proof system [20]. The combination of these two results demonstrates that extended resolution polynomially simulates the $\mathsf {PR}$ proof system and therefore also its restricted variants. An open question is how the $\mathsf {PR}$ proof system without new variables relates to other strong proof systems for propositional logic that do not introduce new variables, such as Frege systems. Other open questions are related to the space and width bounds of the smallest $\mathsf {PR}$ proofs, again without new variables, for well-known other hard problems such as Tseitin formulas [26, 27] or pebbling games [25].

On the practical side, we want to pursue some ideas to improve SAT solving by learning short $\mathsf {PR}$ clauses. Our first approach, called *satisfaction-driven clause learning*, generalizes the well-known conflict-driven clause learning paradigm by checking whether certain assignments—encountered during solving—can be pruned from the search space by adding $\mathsf {PR}$ clauses [15]. Our current implementation can find short proofs of pigeon hole formulas, although the solver can only find a subset of all possible $\mathsf {PR}$ clauses. Moreover, we are still searching for efficient heuristics that help solvers with finding short $\mathsf {PR}$ clauses in general formulas. Another problem we are currently exploring is the minimization of conflict clauses by checking if a subset of a conflict clause is propagation redundant with respect to the formula under consideration. Finally, we want to implement a formally-verified proof checker for $\mathsf {PR}$ proofs.

## Conclusion

We presented a clean and simple characterization of clause redundancy that is based on an implication relation between a formula and itself under different partial assignments. Replacing the implication relation by efficiently decidable notions of implication, e.g., the superset relation or implication via unit propagation, gives then rise to various polynomially-checkable redundancy criteria. One variant yields a proof system that turns out to coincide with $\mathsf {RAT}$, which together with deletion is the de-facto standard in SAT solving. We conjecture the proof systems based on the other two variants to be stronger if the introduction of new variables is not allowed. We showed that these more general proof systems admit short clausal proofs without new variables for the famous pigeon hole formulas. Experiments show that our proofs are much smaller than existing clausal proofs and that they are also much faster to check. Our new proof systems concisely simulate many other concepts from the literature such as autarkies, variable instantiation, safe assignments, and certain kinds of symmetry reasoning.

## Footnotes

1 We changed the definition of $$ PHP _{n}$$ from putting *n* pigeons into $$n-1$$ holes to putting $$n+1$$ pigeons into *n* holes in order to fix a discrepancy with the formulas in the evaluation.

2 In the conference version [14] of this article, we wrongly stated that we can add all *falsified* unit clauses instead of the *satisfied* ones.
